# Surveillance and Diagnosis of West Nile Virus in the Face of Flavivirus Cross-Reactivity

**DOI:** 10.3389/fmicb.2018.02421

**Published:** 2018-10-11

**Authors:** Yaniv Lustig, Danit Sofer, Efrat Dahan Bucris, Ella Mendelson

**Affiliations:** ^1^Central Virology Laboratory, Ministry of Health, Sheba Medical Center, Ramat Gan, Israel; ^2^School of Public Health, Sackler Faculty of Medicine, Tel Aviv University, Tel Aviv, Israel

**Keywords:** WNV, surveillance, one health, diagnosis, Zika, West Nile, mosquitoes, flavivirus

## Abstract

West Nile Virus (WNV) is an arthropod-borne flavivirus whose zoonotic cycle includes both mosquitoes and birds as amplifiers and humans and horses as dead-end hosts. In recent years WNV has been spreading globally and is currently endemic in Africa, The Middle East, India, Australia, central and southern Europe, and the Americas. Integrated surveillance schemes and environmental data aim to detect viral circulation and reduce the risk of infection for the human population emphasizing the critical role for One Health principles in public health. Approximately 20% of WNV infected patients develop West Nile Fever while in less than 1%, infection results in West Nile Neurological Disease. Currently, the diagnosis of WNV infection is primarily based on serology, since molecular identification of WNV RNA is unreliable due to the short viremia. The recent emergence of Zika virus epidemic in America and Asia has added another layer of complexity to WNV diagnosis due to significant cross-reactivity between several members of the Flaviviridae family such as Zika, dengue, Usutu, and West Nile viruses. Diagnosis is especially challenging in persons living in regions with flavivirus co-circulation as well as in travelers from WNV endemic countries traveling to Zika or dengue infected areas or vise-versa. Here, we review the recent studies implementing WNV surveillance of mosquitoes and birds within the One Health initiative. Furthermore, we discuss the utility of novel molecular methods, alongside traditional molecular and serological methods, in WNV diagnosis and epidemiological research.

## Introduction

West Nile Virus (WNV) is a member of the family Flaviviridae from the genus Flavivirus which also contain other viruses pathogenic to humans such as Zika, dengue, yellow fever, Usutu, and Japanese encephalitis (Hayes et al., 2005; Petersen et al., 2013). WNV is maintained in nature in a bird-mosquito cycle with birds acting as amplifying hosts (Malkinson and Banet, 2002). WNV infects mostly mosquitoes from the *Culex* genus which can potentially transmit the virus to every vertebrate on which they feed (Orshan et al., 2008; Andreadis, 2012; Engler et al., 2013; Steiner and Kennedy, 2013; Lustig et al., 2015). While many bird species do not develop any disease, several species such as crows and jays may die of WNV infection (Gamino and Hofle, 2013). Mammals, primarily horses and humans are unable to contribute to the transmission cycle and thus are considered dead end hosts (Colpitts et al., 2012).

West Nile Virus infection in humans is mostly asymptomatic, however, in approximately 20% of the cases WNV infection induces a mild disease with influenza like symptoms termed West Nile Fever (WNF), while in less than 1% of cases, mainly in elderly and immunocompromised people, infection results in a severe neuroinvasive disease (WNND) which may lead to death (Hayes et al., 2005). Horses are more prone to WNV than humans and 10% of infected horses may show neurological signs (Castillo-Olivares and Wood, 2004). Currently, no vaccine or other treatments are available for humans, although inactivated and recombinant vaccines are on the market for horses (Seino et al., 2007).

During the last 20 years WNV has spread geographically and is now common in countries in Africa, Europe, the Middle East, North America, and East Asia where it can cause disease outbreaks (Petersen et al., 2013). While the origin of all WNV lineages and genotypes is most probably Africa (May et al., 2011), WNV is primarily distributed to other areas by bird migration paths (Mackenzie et al., 2004). Such is the case for the introduction of WNV into Europe which is hypothesized to be mediated by birds migrating from Africa to Europe in the spring (Calistri et al., 2010). According to two models, long distance WNV dispersal is dependent on birds while short distance diffusion of the virus is mediated by mosquitoes (Liu et al., 2006; Maidana and Yang, 2009). Other factors contributing to WNV spread and its ability to cause human infection include short- and long-distance migratory species (Rappole and Hubalek, 2003), wind patterns (Mackenzie et al., 2004) and the existence of compatible mosquito vector. Studies in Israel, a major crossroad for bird migration between Africa and Eurasia, have indicated that four different genotypes within two lineages of WNV have been circulating in recent years (Lustig et al., 2015). Due to its complex transmission cycle and lack of treatment, it is now apparent that a multi-disciplinary approach with cross sectorial collaboration between organizations from public, animal and environmental health is needed to obtain knowledge on WNV circulation and prevention of WNV transmission. Unfortunately, WNV is not alone in its global expansion: other flaviviruses such as dengue and Zika have spread or emerged in many countries making the diagnosis of flaviviral diseases substantially more challenging. In this review we will focus on the recent studies implementing WNV surveillance of mosquitoes and birds within the One Health initiative which aim to reduce WNV transmission and discuss the molecular and serological methods available for sensitive and specific WNV diagnosis.

## Wnv Surveillance Within the One Health Initiative

The One Health concept recognizes that the health of humans, animals, and environment are all connected and that only a collaborative inter-disciplinary approach can effectively achieve optimal health outcomes (Lerner and Berg, 2015). WNV is transmitted to humans and animals primarily via the amplification of the mosquitoes – birds’ enzootic cycle which is independently controlled by climatic and environmental factors such as temperatures, seasons and water level fluctuations (Paz and Semenza, 2013). Therefore, active surveillance of WNV in mosquitoes and birds population in combination with analysis of climatic and environmental data offers an opportunity to detect virus prior to the emergence of disease in equine species or human populations and predict timing and locations of future WNV disease outbreaks. Most important, early detection of WNV may facilitate targeted use of insecticides in the infected area to reduce WNV burden in human and animal populations. The necessity of such surveillance schemes is dependent on WNV prevalence and might be implemented in the whole country or restricted to specific region of high WNV endemicity. In the next sections we discuss eight WNV endemic countries which represent several levels of implementation of the One Health concept (**Table 1**).

**Table 1:** West Nile Virus (WNV) incidence in humans and surveillance programs in selected countries.

Country	Disease incidence in humans	Status of surveillance programs	More information (webpage)
**Italy**	Endemic primarily in northern Italy. 173 WNND cases detected in 2008–2015. National incidence and median age for WNND of 1.2/1,000,000 (from 2012 to 2015) and 73 years (2008–2015), respectively (Rizzo et al., 2016).	Integrated surveillance of human, mosquitoes, wild birds, and horses in endemic regions in Italy. Blood donations testing are activated based on surveillance data.	
**Greece**	Outbreaks of WNV infection in 2010–2012 with overall 525 cases. In the 2012 outbreak national incidence of WNND cases varied from 1.7/100,000 in the 30–39 year-olds to 4.22/100,000 in ≥80 years old. Median age was 70 (Pervanidou et al., 2014).	An integrated entomological surveillance is in place. Animal and human WNV cases are notified and data is shared.	http://www.keelpno.gr/en-us/home. aspx
**Spain**	Two human cases in 2010 and 3 in 2016 in Andalusia.	Sporadic surveillance in mosquitoes. Notifiable disease in humans and horses.	
**Austria**	Seventeen human cases detected in 2010–2017.	A national WNV task force integrates data on WNV in animals, mosquitoes and humans.	
**Israel**	Endemic with annual WNV cases ranging between 12 and 429 with a total of 1382 in 2000–2012 (Anis et al., 2014). In the last outbreak in 2015 national incidence of WNF and WNND was 1.8/100,000. Median age was 64.7 in live cases and 75.6 in fatal cases (Salama et al., 2018).	Mosquito and human data are shared routinely. A strategic inter-ministerial program to integrate data from animals, humans, and mosquitoes was recently initiated.	
**Turkey**	Outbreaks of WNV infection in 2010 and 2011 with 47 and 5 cases, respectively.	Sporadic studies on WNV seroprevalence in horses and WNV RNA in mosquitoes.	
**United States**	WNV is present in all 48 continental states. Between 1999 and 2016 21,574 WNND cases were detected. In 2017, 1339 WNND cases were detected. In 1999–2016, national incidence of WNND varied from 0.25/100,000 in the 30–39 year-olds to 1.1/100,000 in ≥70 years old.	National surveillance data capture platform – ArboNET integrates data of WNV in humans, animals and mosquitoes in addition to state specific surveillance programs.	
**Canada**	In 2016 WNV was present in seven provinces with a total of 60 WNND cases. Overall from 2002 to 2016 5614 WNV infection cases were detected.	Canada conducts ongoing surveillance at the national level in humans, animals, and mosquitoes.	


### Italy

Following the first outbreak of WNV in horses in Italy in 1998 (Cantile et al., 2000; Autorino et al., 2002) the Italian government has started in 2001 a national surveillance plan that targeted birds, domestic poultry, horses, mosquitoes, and humans (Angelini et al., 2010). In recent years this plan, which is carried out in three regions in the Po valley area in northern Italy where WNV is endemic, has developed and is now based on the trans disciplinary and trans-sectorial collaboration between regional institutions involved in public, human, animal, and environmental health (Angelini et al., 2010; Mulatti et al., 2013; Napoli et al., 2013; Rosa et al., 2014; Calzolari et al., 2015). The integrated surveillance system also allows to modulate blood donations screening in the Emilia-Romagna region as only WNV detection by the surveillance system triggers public health interventions on blood donors (Paternoster et al., 2017a). To improve the surveillance sensitivity, data sharing mechanisms have been established among the three regions which are aimed at early detection of the viral circulation and reduction of the risk of infection (Paternoster et al., 2017b). Since WNV can be transferable through blood donations, seroprevalence of WNV antibodies among blood donors is also examined (Pezzotti et al., 2011; Pierro et al., 2011, 2013; Gaibani et al., 2013).

### Greece

West Nile Virus has emerged in Greece in 2010 and caused outbreaks for three consecutive years in both humans and horses (Papa et al., 2010; Danis et al., 2011a,b; Papa, 2012; Bouzalas et al., 2016). Following the 2010 outbreak a strategic framework for an integrated entomological surveillance program was established with the aims to address the impact of current vector control strategies and climate on mosquito population, to analyze the mosquito species composition in WNV affected areas as well as other areas of Greece and to quantify viral circulation, geographic spread and transmission cycle of WNV in mosquitoes (Gomes et al., 2013; Valiakos et al., 2014; Patsoula et al., 2016). In recent years seroprevalence studies in both humans (Ladbury et al., 2013; Vrioni et al., 2014), chickens (Chaintoutis et al., 2016), and equine (Bouzalas et al., 2016) were initiated to examine the kinetics of WNV prevalence in animals and humans over time.

### Spain

The number of WNV cases detected in horses and humans in Spain is significantly smaller than Italy and Greece and so far only two outbreaks with two and three human patients have been recorded in Andalusia, in 2010 and 2016, respectively (Garcia-Bocanegra et al., 2011, 2012; Lopez-Ruiz et al., 2018). Nevertheless, monitoring of mosquitoes infected with WNV and screening of WNV seropositivity in humans, horses, and birds in Spain has enabled the detection of WNV circulation years before the occurrence of cases in humans or horses (Figuerola et al., 2007; Lopez et al., 2008; Jimenez-Clavero et al., 2010; Alba et al., 2014). Currently, surveillance of WNV in Spain is primarily investigated as research projects run by universities and other private institutions and not by the Spanish or municipal government and as such is focused on specific years and/or regions. Recently, risk mapping of WNV circulation in 2015 in Spain was conducted with the hope to improve WNV risk-based surveillance and develop a model for WNV distribution and infection in Spain (Sanchez-Gomez et al., 2017).

### Israel

West Nile Virus has been recognized as endemic in Israel since its establishment in 1948. However, despite sporadic cases and a few small outbreaks (Bernkopf et al., 1953; Flatau et al., 1981; Katz et al., 1989), WNV was not considered a public health concern until 2000, when a large scale human outbreak occurred (Bin et al., 2001; Weinberger et al., 2001). Since then outbreaks of varying magnitudes have been recorded every few years (Anis et al., 2014). Following the 2000 outbreak, a national mosquito surveillance system was established which is responsible for entomological analysis of mosquitoes and characterization of WNV circulation in Israel (Orshan et al., 2008; Lustig et al., 2015). Furthermore, WNV infections in humans and seropositivity of the population are monitored routinely and are integrated with mosquito surveillance data by the public health division to assess the burden of WNV circulation in Israel and implement appropriate measures (Lustig et al., 2015, 2017a,b; Bassal et al., 2017). Recently, the public health and veterinary services, Israel Nature and Park Authorities and the ministry of environmental protection have initiated a program to integrate WNV data obtained from humans, mosquitos, horses, and birds with the aim to develop a true one health initiative for WNV in Israel. Finally, testing of blood donors was initiated in 2017 to assess the extent of alternative routes of WNV transmission to humans in Israel.

### Turkey

Much like its neighbor, Greece, WNV outbreaks emerged in 2010 and 2011 with 47 and 5 human cases, respectively (Kalaycioglu et al., 2012). Since then several seroprevalence and mosquito surveillance reports confirmed the presence of WNV in Turkey (Ergunay et al., 2013, 2014, 2015). A recent study was initiated to provide a risk-assessment of the circulation of mosquito borne flaviviruses in Turkey (Ergunay et al., 2017), however, an integrated national surveillance plan is not yet in place.

### Austria

West Nile Virus was first identified in Austria in birds in 2008 (Bakonyi et al., 2013) and human infection has been recorded since 2009 (Stiasny et al., 2013). Since then several WNV clinical cases are diagnosed each year in humans (Gossner et al., 2017; Kolodziejek et al., 2018). In 2013, a national WNV task force was established which is responsible to integrate all data collected from mosquito surveillance systems, veterinary surveillance of birds and horses and the Austrian Blood Donation System and public health authorities (Gossner et al., 2017; Kolodziejek et al., 2018). Altogether, detailed reports of WNV activity in Austria are routinely published and through the combined effort of multiple independently operating agencies WNV activity in Austria is closely monitored (Kolodziejek et al., 2018).

### United States

West Nile Virus had not been detected in North America before 1999, when a large WNV outbreak occurred in New York City (Nash et al., 2001). Phylogenetic studies identified genetic similarity to strains previously identified in Israel, suggesting a Middle Eastern importation (Lanciotti et al., 1999). Since then, the 1999 outbreak strain was rapidly displaced by a novel North American genotype (NA/WN02) (Davis et al., 2005) which can now be found in all 48 contiguous states and was responsible for large nationwide epidemics in 2003 and 2012 (Arnold, 2012; Roehrig, 2013). WNV can be transmitted under lab setting (Turell et al., 2005; Vanlandingham et al., 2007) and in nature (Andreadis et al., 2001; Barker et al., 2009) by several mosquito species circulating in the United States. In addition to state specific mosquito surveillance programs (White et al., 2001; Barker et al., 2003; Tesh et al., 2004; Poh et al., 2018), the Centers for disease control and prevention (CDC) has established in 2000 a comprehensive and robust national surveillance data capture platform termed ArboNET in order to monitor WNV patterns in humans, mosquitoes, birds, and other animals and track the progression of WNV activity across the United States (Hadler et al., 2015). The use of ArboNET, state specific arboviral surveillance systems and environmental monitoring has allowed prediction of a WNV outbreak in the Great Plains, an area with high levels of WNV circulation (Chuang and Wimberly, 2012; Davis et al., 2017). However, despite association of WNV outbreaks in the United States with parameters such as urban and ecological habitats (Bowden et al., 2011), rural irrigated landscapes (DeGroote and Sugumaran, 2012), increased temperature (Hartley et al., 2012), several socioeconomic factors such as housing age and community drainage patterns (Ruiz et al., 2007), per capita income (DeGroote and Sugumaran, 2012), and neglected swimming pool density (Reisen et al., 2008; Harrigan et al., 2010), no models have been developed that predict how these factors combine to produce outbreaks. Since the Zika virus outbreak in 2015, ArboNET is routinely used for Zika disease reporting, thus allowing the quick integration and dissemination of data across the United States (Simeone et al., 2016).

### Canada

Following WNV introduction to the United States, the virus has spread to Canada and was responsible for major outbreaks in 2002 and 2012 in Ontario and Quebec and in 2003 and 2007 in the Prairie Provinces (Zheng et al., 2014; Kulkarni et al., 2015). In order to cope with the disease, integrated mosquito surveillance is routinely performed at the national level and WNV disease is nationally notifiable and reportable (Zheng et al., 2014). In addition, surveillance data on humans, horses, and birds is shared between all municipalities and blood donations are routinely checked for WNV RNA presence. Data assembly into weekly national reports and studies evaluating the dynamics of WNV transmission using human case prevalence, mosquito surveillance, and climate data (Chen et al., 2013; Giordano et al., 2017; Mallya et al., 2018) contribute to raising the awareness of WNV in Canada.

### Assessment of One Health Initiatives

At this point it is hard to assess the effects that One Health initiatives have on controlling WNV disease in each country. In Italy, an unbiased quantitative evaluation protocol has been developed to examine the implementation of the One Health approach in order to “fine tune” the system (Paternoster et al., 2017b). However, since One Health is a relatively young approach more time in needed to retrospectively examine its effect on reduction of WNV circulation and infection.

## Wnv Rna Diagnosis

With the development of molecular biology methods in the 1980s, laboratory diagnosis of viral infections have developed from traditional viral isolation in cell culture which can take days to weeks (Leland and Ginocchio, 2007) to molecular detection of viral genomes which can be achieved in hours. Acute WNV infection in humans can be diagnosed by WNV RNA detection in samples obtained from symptomatic patients. In addition, since WNV can be transferable through blood (Pealer et al., 2003; Montgomery et al., 2006), screening of blood and organ donations obtained from persons living in WNV endemic areas is important to identify samples that are infected with WNV (Pisani et al., 2016).

### Diagnosis of WNV Acute Infection

**FIGURE 1:**
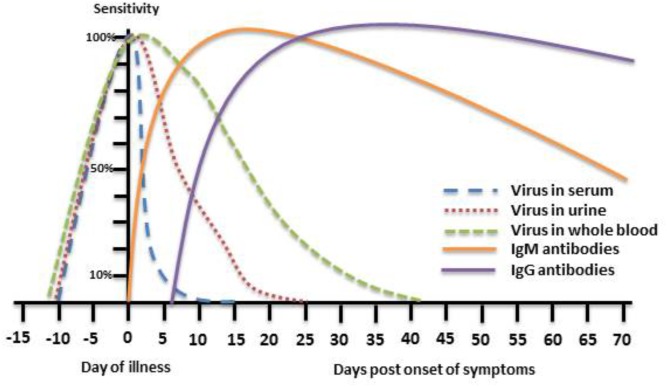
Theoretical depiction of WNV presence in body fluids and WNV immune response. The phases of WNV detection period in serum, urine, and whole blood as well as the IgM and IgG immune response to WNV infection are presented with respect to the day of illness.

In recent years, the detection of viral genome in bodily fluids by quantitative Real-Time Polymerase Chain Reaction (qRT-PCR) has become the routine diagnosis method for many viral infections due to its industry standard format, high levels of repeatability and reproducibility, high sensitivity and specificity, fast turnaround time and ease of use (Boonham et al., 2014). Unfortunately, for most diseases caused by flaviviruses, including WNF, molecular diagnosis by qRT-PCR of serum, plasma and cerebrospinal fluid (CSF) samples is of limited value for routine diagnosis due to low level and short lived viremia generated by these viruses (Busch et al., 2006, 2008; Barzon et al., 2013a; Lustig et al., 2016; **Figure 1**). Recently, several studies have demonstrated that WNV is retained in the kidneys and can therefore be detected in urine samples for a longer period of time than plasma, CSF or serum (Barzon et al., 2013a,b, 2014, 2015). In addition, since WNV was shown to adhere to red blood cells (Rios et al., 2007) it can persist for months in whole blood of blood donors (Lanteri et al., 2014). We have recently found that during acute WNV infection WNV RNA can be detected in whole blood, serum, CSF, plasma, and urine samples in 86.8, 26, 16.6, 20 and 58.3% of WNV infected patients, respectively, demonstrating the superiority and effectiveness of WNV RNA detection in whole blood for diagnosis of acute Infection (Lustig et al., 2016; **Figure 1**). Several commercial and in house molecular diagnostic tests with varying sensitivities are available which are different primarily in their amplification target of the viral genome.

### Blood and Organ Screening

Transmission of WNV during blood and organ donations has been recorded in the United States (Gyure, 2009) and Europe (Morelli et al., 2010; Costa et al., 2011; Rabel et al., 2011; Inojosa et al., 2012). As mentioned previously, 80% of WNV infections in humans are asymptomatic and most persons are not aware they have been infected with WNV, especially in areas endemic for WNV (Hayes et al., 2005). Therefore, screening of blood donations for WNV is performed annually in many countries with WNV endemic circulation, such as the United States, Canada, several countries in the European Union and Israel to assess and reduce the risk of organ transplant and blood recipients for infection with WNV. A few commercial nucleic acid amplification tests (NATs) are on the market which are approved for blood testing from healthy persons (Pai et al., 2008; Ziermann and Sanchez-Guerrero, 2008; Zhang et al., 2009), are fully automated, and allow testing on hundreds of plasma samples a day. Due to the low levels of WNV RNA that may be present in healthy, asymptomatic blood donors, these commercial tests need to detect WNV with very high sensitivity and specificity.

## Serological Wnv Diagnosis

Due to the challenges with WNV viral detection, specific antibody testing is currently the most widely used approach for WNV diagnosis. Immunoglobulin M (IgM) and Immunoglobulin G (IgG) antibodies can usually be detected by day 4 and day 8 after onset of symptoms, respectively (Busch et al., 2008) and therefore detection of IgM antibodies alone or IgG seroconversion can point to a WNV acute infection (**Figure 1**). Numerous commercial kits for WNV antibody detection are available which are primarily based on detection of specific antibodies against the Envelope (E) protein of WNV using either enzyme linked immunosorbent assays (ELISAs) or immunofluorescence tests (IFAs). The advantages of these qualitative assays are that they are very easy to use in a laboratory setting, the results are mostly reproducible and part of their protocol can be automated. However, since WNV IgM can persist in serum for months and even years after infection (Prince et al., 2005; Busch et al., 2008; Murray et al., 2010, 2013; Papa et al., 2011, 2015) and significant cross reactivity exists between all flaviviruses it is often difficult to diagnose acute WNV infection based on these commercial kits alone. To validate the initial results neutralization assays can be used. Neutralization determines the ability of the antibodies present in the patient’s sample to neutralize the virus and therefore it can evaluate the antibody-virus neutralization efficiency (Nelson et al., 2008) and is considered the gold standard for diagnosis of WNV infection (Calisher et al., 1989; Kuno, 2003). There are several types of neutralization protocols with similar performance which differ in their sensitivity [plaque reduction neutralization (PRNT) 50 or PRNT90], the method of cytopathic effect (CPE) detection (direct, staining, detection with IFA) and the plates used (for PRNT or micro-neutralization).

The Zika epidemic, which emerged in 2015 in Brazil (Fauci and Morens, 2016; Song et al., 2017), poses another layer of complication for diagnosis of flaviviruses, including WNV. Due to the high cross-reactivity of Zika virus with other flavivirus antibodies, E protein based ELISAs and neutralization assays encounter difficulties to distinguish between specific flavivirus infections and as a result diagnosis of WNV or dengue virus (Lanciotti et al., 2008; Balmaseda et al., 2017) in persons with Zika virus background is more challenging. Our experience (unpublished results) with Zika positive patients show high cross reactivity with WNV E protein based ELISAs which can cause false positive results for encephalitic patients and as a consequence the wrong treatment. The development of ELISA tests for Zika infection which use the Non-structural 1 (NS1) protein as antigen for Zika antibody testing proved to be advantageous and more specific than E protein based ELISAs (Steinhagen et al., 2016), however, sensitivity and detection times issues, especially in countries with previous dengue (Steinhagen et al., 2016) and possibly WNV (Lustig et al., 2017c) background limit the dependence on this test. Development of similar NS1 antibody based ELISA assays for WNV and dengue virus could be useful in reducing the cross-reactivity resulting in more specific Flavivirus diagnostic tests.

## Novel Methods for Wnv Detection

Serological methods aided by detection of viral nucleic acid by qRT-PCR will, most probably, continue to be the primary and preferred diagnostic methods for WNV. Nevertheless, the establishment of next generation sequencing (NGS) methods in recent years, which are also called deep or high-throughput sequencing, led to the increased use of NGS to complement diagnosis and monitoring of infectious diseases caused by both bacteria (Deurenberg et al., 2017) and viruses (Hoper et al., 2016; Casadella and Paredes, 2017; Ramamurthy et al., 2017). The most important diagnostic application for NGS is probably the unbiased identification of pathogens in clinical samples. With regards to WNV, one example is the identification of WNV by NGS from a CSF sample obtained from a 14 years old meningoencephalitis patient (Wilson et al., 2017). In a recent study, plasma samples from 12 cases of unexplained febrile illness in Tanzania were investigated by NGS and identified WNV in two samples (Williams et al., 2018). These examples demonstrate the additive value that NGS may provide even in diagnosis of viruses with short viremia such as WNV. NGS can also be used in epidemiological investigations and research (Zana et al., 2016).

Recently, a new and novel method for the detection of flavivirus RNAs including Zika, dengue and WNV from body fluids has emerged. Using CRISPR-cas13 technology, a fluorescent protein was used to track and detect flavivirus RNAs present in patients samples generating a field-deployable viral diagnostic platform with high performance and minimal equipment or sample processing requirements (Myhrvold et al., 2018). This platform is comparable in its sensitivity to qRT-PCR assays, is fast (under 2 h) and may be used to serve as a diagnostic platform in areas with limited resources or even compete with current molecular diagnostic devices which are in the market.

## Concluding Remarks

The emergence and geographical spread of WNV in recent years has forced endemic countries to initiate integrated surveillance programs to identify, predict, and reduce WNV circulation. Future programs should be implemented to combine all data and assess the effectiveness of these surveillance programs to reduce the burden of WNV infections. In addition, due to the influx of other flaviviruses, such as Zika virus, and their global expansion, more specific, yet sensitive assays for WNV diagnosis should be established to be able to distinguish between the different diseases and ultimately pave the way for the development of a WNV vaccine.

## Author Contributions

YL reviewed the design, researched the literature, and wrote and edited the manuscript. DS researched the literature and edited and provided critical review of the manuscript. EB researched the literature and wrote and edited the manuscript. EM reviewed the design, researched the literature, and edited and provided critical review of the manuscript.

## Conflict of Interest Statement

The authors declare that the research was conducted in the absence of any commercial or financial relationships that could be construed as a potential conflict of interest.

## References

[B1] Alba A. Allepuz A. Napp S. Soler M. Selga I. Aranda C. (2014). Ecological surveillance for West Nile in Catalonia (Spain), learning from a five-year period of follow-up. *Zoon. Public Health* 61 181–191. 10.1111/zph.12048 23590452

[B2] Andreadis T. G. (2012). The contribution of Culex pipiens complex mosquitoes to transmission and persistence of West Nile virus in North America. *J. Am. Mosq. Control Assoc.* 28 137–151. 23401954 10.2987/8756-971X-28.4s.137

[B3] Andreadis T. G. Anderson J. F. Vossbrinck C. R. (2001). Mosquito surveillance for West Nile virus in Connecticut, 2000: isolation from *Culex pipiens*, *Cx. restuans*, *Cx. salinarius*, and *Culiseta melanura*. *Emerg. Infect. Dis.* 7 670–674. 11585530 10.3201/eid0704.010413 PMC2631746

[B4] Angelini P. Tamba M. Finarelli A. C. Bellini R. Albieri A. Bonilauri P. (2010). West Nile virus circulation in Emilia-Romagna, Italy: the integrated surveillance system 2009. *Euro. Surveill.* 15:19547. 20430000

[B5] Anis E. Grotto I. Mendelson E. Bin H. Orshan L. Gandacu D. (2014). West Nile fever in Israel: the reemergence of an endemic disease. *J. Infect.* 68 170–175. 10.1016/j.jinf.2013.10.009 24183889

[B6] Arnold C. (2012). West Nile virus bites back. *Lancet Neurol.* 11 1023–1024. 23153403 10.1016/S1474-4422(12)70278-8

[B7] Autorino G. L. Battisti A. Deubel V. Ferrari G. Forletta R. Giovannini A. (2002). West Nile virus epidemic in horses, Tuscany region, Italy. *Emerg. Infect. Dis.* 8 1372–1378. 12498650 10.3201/eid0812.020234 PMC2738505

[B8] Bakonyi T. Ferenczi E. Erdelyi K. Kutasi O. Csorgo T. Seidel B. (2013). Explosive spread of a neuroinvasive lineage 2 West Nile virus in Central Europe, 2008/2009. *Vet. Microbiol.* 165 61–70. 10.1016/j.vetmic.2013.03.005 23570864

[B9] Balmaseda A. Stettler K. Medialdea-Carrera R. Collado D. Jin X. Zambrana J. V. (2017). Antibody-based assay discriminates Zika virus infection from other flaviviruses. *Proc. Natl. Acad. Sci. U.S.A.* 114 8384–8389. 10.1073/pnas.1704984114 28716913 PMC5547631

[B10] Barker C. M. Bolling B. G. Black W. C. T. Moore C. G. Eisen L. (2009). Mosquitoes and West Nile virus along a river corridor from prairie to montane habitats in eastern Colorado. *J. Vector Ecol.* 34 276–293. 10.1111/j.1948-7134.2009.00036.x 20836831

[B11] Barker C. M. Reisen W. K. Kramer V. L. (2003). California state mosquito-borne virus surveillance and response plan: a retrospective evaluation using conditional simulations. *Am. J. Trop. Med. Hyg.* 68 508–518. 12812335 10.4269/ajtmh.2003.68.508

[B12] Barzon L. Pacenti M. Franchin E. Pagni S. Martello T. Cattai M. (2013a). Excretion of West Nile virus in urine during acute infection. *J. Infect. Dis.* 208 1086–1092. 10.1093/infdis/jit290 23821721

[B13] Barzon L. Pacenti M. Franchin E. Squarzon L. Sinigaglia A. Ulbert S. (2014). Isolation of West Nile virus from urine samples of patients with acute infection. *J. Clin. Microbiol.* 52 3411–3413. 10.1128/JCM.01328-14 24951801 PMC4313171

[B14] Barzon L. Pacenti M. Palu G. (2013b). West Nile virus and kidney disease. *Expert. Rev. Anti Infect. Ther.* 11 479–487. 10.1586/eri.13.34 23627854

[B15] Barzon L. Pacenti M. Ulbert S. Palu G. (2015). Latest developments and challenges in the diagnosis of human West Nile virus infection. *Expert. Rev. Anti Infect. Ther.* 13 327–342. 10.1586/14787210.2015.1007044 25641365

[B16] Bassal R. Shohat T. Kaufman Z. Mannasse B. Shinar E. Amichay D. (2017). The seroprevalence of West Nile Virus in Israel: a nationwide cross sectional study. *PLoS One* 12:e0179774. 10.1371/journal.pone.0179774 28622360 PMC5473576

[B17] Bernkopf H. Levine S. Nerson R. (1953). Isolation of West Nile virus in Israel. *J. Infect. Dis.* 93 207–218. 13109233 10.1093/infdis/93.3.207

[B18] Bin H. Grossman Z. Pokamunski S. Malkinson M. Weiss L. Duvdevani P. (2001). West Nile fever in Israel 1999-2000: from geese to humans. *Ann. N. Y. Acad. Sci.* 951 127–142. 11797770 10.1111/j.1749-6632.2001.tb02691.x

[B19] Boonham N. Kreuze J. Winter S. van der Vlugt R. Bergervoet J. Tomlinson J. (2014). Methods in virus diagnostics: from ELISA to next generation sequencing. *Virus Res.* 186 20–31. 10.1016/j.virusres.2013.12.007 24361981

[B20] Bouzalas I. G. Diakakis N. Chaintoutis S. C. Brellou G. D. Papanastassopoulou M. Danis K. (2016). Emergence of Equine West Nile Encephalitis in Central Macedonia, Greece, 2010. *Transbound Emerg. Dis.* 63 e219–e227. 10.1111/tbed.12334 25660661

[B21] Bowden S. E. Magori K. Drake J. M. (2011). Regional differences in the association between land cover and West Nile virus disease incidence in humans in the United States. *Am. J. Trop. Med. Hyg.* 84 234–238. 10.4269/ajtmh.2011.10-0134 21292890 PMC3029173

[B22] Busch M. P. Kleinman S. H. Tobler L. H. Kamel H. T. Norris P. J. Walsh I. (2008). Virus and antibody dynamics in acute west nile virus infection. *J. Infect. Dis.* 198 984–993. 10.1086/591467 18729783

[B23] Busch M. P. Wright D. J. Custer B. Tobler L. H. Stramer S. L. Kleinman S. H. (2006). West Nile virus infections projected from blood donor screening data, United States, 2003. *Emerg. Infect. Dis.* 12 395–402. 16704775 10.3201/eid1203.051287 PMC3291460

[B24] Calisher C. H. Karabatsos N. Dalrymple J. M. Shope R. E. Porterfield J. S. Westaway E. G. (1989). Antigenic relationships between flaviviruses as determined by cross-neutralization tests with polyclonal antisera. *J. Gen. Virol.* 70(Pt 1) 37–43. 2543738 10.1099/0022-1317-70-1-37

[B25] Calistri P. Giovannini A. Hubalek Z. Ionescu A. Monaco F. Savini G. (2010). Epidemiology of west nile in europe and in the mediterranean basin. *Open Virol. J.* 4 29–37. 10.2174/1874357901004020029 20517490 PMC2878979

[B26] Calzolari M. Pautasso A. Montarsi F. Albieri A. Bellini R. Bonilauri P. (2015). West Nile Virus Surveillance in 2013 via mosquito screening in northern Italy and the influence of weather on virus circulation. *PLoS One* 10:e0140915. 10.1371/journal.pone.0140915 26488475 PMC4619062

[B27] Cantile C. Di Guardo G. Eleni C. Arispici M. (2000). Clinical and neuropathological features of West Nile virus equine encephalomyelitis in Italy. *Equine Vet. J.* 32 31–35. 10661382 10.2746/042516400777612080

[B28] Casadella M. Paredes R. (2017). Deep sequencing for HIV-1 clinical management. *Virus Res.* 239 69–81. 10.1016/j.virusres.2016.10.019 27818211

[B29] Castillo-Olivares J. Wood J. (2004). West Nile virus infection of horses. *Vet. Res.* 35 467–483. 15236677 10.1051/vetres:2004022

[B30] Chaintoutis S. C. Gewehr S. Mourelatos S. Dovas C. I. (2016). Serological monitoring of backyard chickens in Central Macedonia-Greece can detect low transmission of West Nile virus in the absence of human neuroinvasive disease cases. *Acta Trop.* 163 26–31. 10.1016/j.actatropica.2016.07.018 27469618

[B31] Chen C. C. Epp T. Jenkins E. Waldner C. Curry P. S. Soos C. (2013). Modeling monthly variation of Culex tarsalis (Diptera: Culicidae) abundance and West Nile Virus infection rate in the Canadian Prairies. *Int. J. Environ. Res. Public Health* 10 3033–3051. 10.3390/ijerph10073033 23880728 PMC3734475

[B32] Chuang T. W. Wimberly M. C. (2012). Remote sensing of climatic anomalies and West Nile virus incidence in the northern Great Plains of the United States. *PLoS One* 7:e46882. 10.1371/journal.pone.0046882 23071656 PMC3465277

[B33] Colpitts T. M. Conway M. J. Montgomery R. R. Fikrig E. (2012). West Nile Virus: biology, transmission, and human infection. *Clin. Microbiol. Rev.* 25 635–648. 10.1128/CMR.00045-12 23034323 PMC3485754

[B34] Costa A. N. Capobianchi M. R. Ippolito G. Palu G. Barzon L. Piccolo G. (2011). West Nile virus: the Italian national transplant network reaction to an alert in the north-eastern region, Italy 2011. *Euro. Surveill.* 16:19991. 22008198

[B35] Davis C. T. Ebel G. D. Lanciotti R. S. Brault A. C. Guzman H. Siirin M. (2005). Phylogenetic analysis of North American West Nile virus isolates, 2001-2004: evidence for the emergence of a dominant genotype. *Virology* 342 252–265. 16137736 10.1016/j.virol.2005.07.022

[B36] Danis K. Papa A. Papanikolaou E. Dougas G. Terzaki I. Baka A. (2011a). Ongoing outbreak of West Nile virus infection in humans, Greece, July to August 2011. *Euro. Surveill.* 16:19951. 21903037

[B37] Danis K. Papa A. Theocharopoulos G. Dougas G. Athanasiou M. Detsis M. (2011b). Outbreak of West Nile virus infection in Greece, 2010. *Emerg. Infect. Dis.* 17 1868–1872. 22000357 10.3201/eid1710.110525 PMC3310677

[B38] Davis J. K. Vincent G. Hildreth M. B. Kightlinger L. Carlson C. Wimberly M. C. (2017). Integrating Environmental monitoring and mosquito surveillance to predict vector-borne disease: prospective forecasts of a west nile virus outbreak. *PLoS Curr.Outbreaks*. 10.1371/currents.outbreaks.90e80717c4e67e1a830f17feeaaf85de 28736681 PMC5503719

[B39] DeGroote J. P. Sugumaran R. (2012). National and regional associations between human West Nile virus incidence and demographic, landscape, and land use conditions in the coterminous United States. *Vector Borne Zoonotic Dis.* 12 657–665. 10.1089/vbz.2011.0786 22607071

[B40] Deurenberg R. H. Bathoorn E. Chlebowicz M. A. Couto N. Ferdous M. Garcia-Cobos S. (2017). Application of next generation sequencing in clinical microbiology and infection prevention. *J. Biotechnol.* 243 16–24. 10.1016/j.jbiotec.2016.12.022 28042011

[B41] Engler O. Savini G. Papa A. Figuerola J. Groschup M. H. Kampen H. (2013). European surveillance for West Nile virus in mosquito populations. *Int. J. Environ. Res. Public Health* 10 4869–4895. 10.3390/ijerph10104869 24157510 PMC3823308

[B42] Ergunay K. Bakonyi T. Nowotny N. Ozkul A. (2015). Close relationship between West Nile virus from Turkey and lineage 1 strain from Central African Republic. *Emerg. Infect. Dis.* 21 352–355. 10.3201/eid2102.141135 25625703 PMC4313653

[B43] Ergunay K. Gunay F. Erisoz Kasap O. Oter K. Gargari S. Karaoglu T. (2014). Serological, molecular and entomological surveillance demonstrates widespread circulation of West Nile virus in Turkey. *PLoS Negl. Trop. Dis.* 8:e3028. 10.1371/journal.pntd.0003028 25058465 PMC4109882

[B44] Ergunay K. Gunay F. Oter K. Kasap O. E. Orsten S. Akkutay A. Z. (2013). Arboviral surveillance of field-collected mosquitoes reveals circulation of West Nile virus lineage 1 strains in Eastern Thrace, Turkey. *Vector Borne Zoon. Dis.* 13 744–752. 10.1089/vbz.2012.1288 23919608

[B45] Ergunay K. Litzba N. Brinkmann A. Gunay F. Sarikaya Y. Kar S. (2017). Co-circulation of West Nile virus and distinct insect-specific flaviviruses in Turkey. *Parasit. Vectors* 10:149. 10.1186/s13071-017-2087-7 28320443 PMC5360070

[B46] Fauci A. S. Morens D. M. (2016). Zika Virus in the Americas–Yet Another Arbovirus Threat. *N. Engl. J. Med.* 374 601–604. 26761185 10.1056/NEJMp1600297

[B47] Figuerola J. Soriguer R. Rojo G. Gomez Tejedor C. Jimenez-Clavero M. A. (2007). Seroconversion in wild birds and local circulation of West Nile virus, Spain. *Emerg. Infect. Dis.* 13 1915–1917. 10.3201/eid1312.070343 18258046 PMC2876749

[B48] Flatau E. Kohn D. Daher O. Varsano N. (1981). West Nile fever encephalitis. *Isr. J. Med. Sci.* 17 1057–1059. 6274825

[B49] Gaibani P. Pierro A. Lunghi G. Farina C. Toschi V. Matinato C. (2013). Seroprevalence of West Nile virus antibodies in blood donors living in the metropolitan area of Milan, Italy, 2009-2011. *New Microbiol.* 36 81–83. 23435819

[B50] Gamino V. Hofle U. (2013). Pathology and tissue tropism of natural West Nile virus infection in birds: a review. *Vet. Res.* 44:39. 10.1186/1297-9716-44-39 23731695 PMC3686667

[B51] Garcia-Bocanegra I. Arenas-Montes A. Napp S. Jaen-Tellez J. A. Fernandez-Morente M. Fernandez-Molera V. (2012). Seroprevalence and risk factors associated to West Nile virus in horses from Andalusia, Southern Spain. *Vet. Microbiol.* 160 341–346. 10.1016/j.vetmic.2012.06.027 22776513

[B52] Garcia-Bocanegra I. Jaen-Tellez J. A. Napp S. Arenas-Montes A. Fernandez-Morente M. Fernandez-Molera V. (2011). West Nile fever outbreak in horses and humans, Spain, 2010. *Emerg. Infect. Dis.* 17 2397–2399. 10.3201/eid1712.110651 22172565 PMC3311180

[B53] Giordano B. V. Kaur S. Hunter F. F. (2017). West Nile virus in Ontario, Canada: a twelve-year analysis of human case prevalence, mosquito surveillance, and climate data. *PLoS One* 12:e0183568. 10.1371/journal.pone.0183568 28829827 PMC5568768

[B54] Gomes B. Kioulos E. Papa A. Almeida A. P. Vontas J. Pinto J. (2013). Distribution and hybridization of Culex pipiens forms in Greece during the West Nile virus outbreak of 2010. *Infect. Genet. Evol.* 16 218–225. 10.1016/j.meegid.2013.02.006 23466890

[B55] Gossner C. M. Marrama L. Carson M. Allerberger F. Calistri P. Dilaveris D. (2017). West Nile virus surveillance in Europe: moving towards an integrated animal-human-vector approach. *Euro. Surveill.* 22:30526. 28494844 10.2807/1560-7917.ES.2017.22.18.30526 PMC5434877

[B56] Gyure K. A. (2009). West Nile virus infections. *J. Neuropathol. Exp. Neurol.* 68 1053–1060. 10.1097/NEN.0b013e3181b88114 19918117

[B57] Hadler J. L. Patel D. Nasci R. S. Petersen L. R. Hughes J. M. Bradley K. (2015). Assessment of Arbovirus Surveillance 13 Years after Introduction of West Nile Virus, United States. *Emerg. Infect. Dis.* 21 1159–1166. 10.3201/eid2107.140858 26079471 PMC4480376

[B58] Harrigan R. J. Thomassen H. A. Buermann W. Cummings R. F. Kahn M. E. Smith T. B. (2010). Economic conditions predict prevalence of West Nile virus. *PLoS One* 5:e15437. 10.1371/journal.pone.0015437 21103053 PMC2980475

[B59] Hartley D. M. Barker C. M. Le Menach A. Niu T. Gaff H. D. Reisen W. K. (2012). Effects of temperature on emergence and seasonality of West Nile virus in California. *Am. J. Trop. Med. Hyg.* 86 884–894. 10.4269/ajtmh.2012.11-0342 22556092 PMC3335698

[B60] Hayes E. B. Sejvar J. J. Zaki S. R. Lanciotti R. S. Bode A. V. Campbell G. L. (2005). Virology, pathology, and clinical manifestations of West Nile virus disease. *Emerg. Infect. Dis.* 11 1174–1179. 16102303 10.3201/eid1108.050289b PMC3320472

[B61] Hoper D. Mettenleiter T. C. Beer M. (2016). Metagenomic approaches to identifying infectious agents. *Rev. Sci. Tech.* 35 83–93. 10.20506/rst.35.1.2419 27217170

[B62] Inojosa W. O. Scotton P. G. Fuser R. Giobbia M. Paolin A. Maresca M. C. (2012). West Nile virus transmission through organ transplantation in north-eastern Italy: a case report and implications for pre-procurement screening. *Infection* 40 557–562. 10.1007/s15010-012-0263-4 22544764

[B63] Jimenez-Clavero M. A. Llorente F. Sotelo E. Soriguer R. Gomez-Tejedor C. Figuerola J. (2010). West Nile virus serosurveillance in horses in Donana, Spain, 2005 to 2008. *Vet. Rec.* 167 379–380. 20817900 10.1136/vr.c3155

[B64] Kalaycioglu H. Korukluoglu G. Ozkul A. Oncul O. Tosun S. Karabay O. (2012). Emergence of West Nile virus infections in humans in Turkey, 2010 to 2011. *Euro. Surveill.* 17:20182. 22687827

[B65] Katz G. Rannon L. Nili E. Danon Y. L. (1989). West Nile fever–occurrence in a new endemic site in the Negev. *Isr. J. Med. Sci.* 25 39–41. 2538406

[B66] Kolodziejek J. Jungbauer C. Aberle S. W. Allerberger F. Bago Z. Camp J. V. (2018). Integrated analysis of human-animal-vector surveillance: west Nile virus infections in Austria, 2015-2016. *Emerg. Microbes Infect.* 7:25. 10.1038/s41426-018-0021-5 29535293 PMC5849732

[B67] Kulkarni M. A. Berrang-Ford L. Buck P. A. Drebot M. A. Lindsay L. R. Ogden N. H. (2015). Major emerging vector-borne zoonotic diseases of public health importance in Canada. *Emerg. Microbes Infect.* 4:e33. 10.1038/emi.2015.33 PMC4773043 26954882

[B68] Kuno G. (2003). Serodiagnosis of flaviviral infections and vaccinations in humans. *Adv. Virus Res.* 61 3–65. 14714429 10.1016/s0065-3527(03)61001-8

[B69] Ladbury G. A. Gavana M. Danis K. Papa A. Papamichail D. Mourelatos S. (2013). Population seroprevalence study after a West Nile virus lineage 2 epidemic, Greece, 2010. *PLoS One* 8:e80432. 10.1371/journal.pone.0080432 24260390 PMC3832368

[B70] Lanciotti R. S. Kosoy O. L. Laven J. J. Velez J. O. Lambert A. J. Johnson A. J. (2008). Genetic and serologic properties of Zika virus associated with an epidemic, Yap State, Micronesia, 2007. *Emerg. Infect. Dis.* 14 1232–1239. 10.3201/eid1408.080287 18680646 PMC2600394

[B71] Lanciotti R. S. Roehrig J. T. Deubel V. Smith J. Parker M. Steele K. (1999). Origin of the West Nile virus responsible for an outbreak of encephalitis in the northeastern United States. *Science* 286 2333–2337. 10600742 10.1126/science.286.5448.2333

[B72] Lanteri M. C. Lee T. H. Wen L. Kaidarova Z. Bravo M. D. Kiely N. E. (2014). West Nile virus nucleic acid persistence in whole blood months after clearance in plasma: implication for transfusion and transplantation safety. *Transfusion* 54 3232–3241. 10.1111/trf.12764 24965017 PMC4268370

[B73] Leland D. S. Ginocchio C. C. (2007). Role of cell culture for virus detection in the age of technology. *Clin. Microbiol. Rev.* 20 49–78. 17223623 10.1128/CMR.00002-06 PMC1797634

[B74] Lerner H. Berg C. (2015). The concept of health in One Health and some practical implications for research and education: what is One Health? *Infect. Ecol. Epidemiol.* 5:25300. 10.3402/iee.v5.25300 25660757 PMC4320999

[B75] Liu R. Shuai J. Wu J. Zhu H. (2006). Modeling spatial spread of west nile virus and impact of directional dispersal of birds. *Math. Biosci. Eng.* 3 145–160. 20361815 10.3934/mbe.2006.3.145

[B76] Lopez G. Jimenez-Clavero M. A. Tejedor C. G. Soriguer R. Figuerola J. (2008). Prevalence of West Nile virus neutralizing antibodies in Spain is related to the behavior of migratory birds. *Vector Borne Zoon. Dis.* 8 615–621. 10.1089/vbz.2007.0200 18399777

[B77] Lopez-Ruiz N. Montano-Remacha M. D. C. Duran-Pla E. Perez-Ruiz M. Navarro-Mari J. M. Salamanca-Rivera C. (2018). West Nile virus outbreak in humans and epidemiological surveillance, west Andalusia, Spain, 2016. *Euro. Surveill.* 23:17–00261. 10.2807/1560-7917.ES.2018.23.14.17-00261 PMC5894251 29637890

[B78] Lustig Y. Hindiyeh M. Orshan L. Weiss L. Koren R. Katz-Likvornik S. (2015). Fifteen years of mosquito surveillance reveals high genetic diversity of West Nile Virus in Israel. *J. Infect. Dis.* 213 1107–1114. 26597260 10.1093/infdis/jiv556

[B79] Lustig Y. Kaufman Z. Mannasse B. Koren R. Katz-Likvornik S. Orshan L. (2017a). West Nile virus outbreak in Israel in 2015: phylogenetic and geographic characterization in humans and mosquitoes. *Clin. Microbiol. Infect.* 23 986–993. 10.1016/j.cmi.2017.04.023 28487165

[B80] Lustig Y. Mannasse B. Koren R. Katz-Likvornik S. Hindiyeh M. Mandelboim M. (2016). Superiority of West Nile Virus RNA Detection in Whole Blood for Diagnosis of Acute Infection. *J. Clin. Microbiol.* 54 2294–2297. 10.1128/JCM.01283-16 27335150 PMC5005505

[B81] Lustig Y. Kaufman Z. Mendelson E. Orshan L. Anis E. Glazer Y. (2017b). Spatial distribution of West Nile virus in humans and mosquitoes in Israel, 2000-2014. *Int. J. Infect. Dis.* 64 20–26. 10.1016/j.ijid.2017.08.011 28882664

[B82] Lustig Y. Zelena H. Venturi G. Van Esbroeck M. Rothe C. Perret C. (2017c). Sensitivity and Kinetics of an NS1-Based Zika Virus Enzyme-Linked Immunosorbent Assay in Zika Virus-Infected Travelers from Israel, the Czech Republic, Italy, Belgium, Germany, and Chile. *J. Clin. Microbiol.* 55 1894–1901. 10.1128/JCM.00346-17 28381608 PMC5442546

[B83] Mackenzie J. S. Gubler D. J. Petersen L. R. (2004). Emerging flaviviruses: the spread and resurgence of Japanese encephalitis, West Nile and dengue viruses. *Nat. Med.* 10 S98–S109. 15577938 10.1038/nm1144

[B84] Maidana N. A. Yang H. M. (2009). Spatial spreading of West Nile Virus described by traveling waves. *J. Theor. Biol.* 258 403–417. 10.1016/j.jtbi.2008.12.032 19167405

[B85] Malkinson M. Banet C. (2002). The role of birds in the ecology of West Nile virus in Europe and Africa. *Curr. Top. Microbiol. Immunol.* 267 309–322. 12082995 10.1007/978-3-642-59403-8_15

[B86] Mallya S. Sander B. Roy-Gagnon M. H. Taljaard M. Jolly A. Kulkarni M. A. (2018). Factors associated with human West Nile virus infection in Ontario: a generalized linear mixed modelling approach. *BMC Infect. Dis.* 18:141. 10.1186/s12879-018-3052-6 29587649 PMC5872497

[B87] May F. J. Davis C. T. Tesh R. B. Barrett A. D. (2011). Phylogeography of West Nile virus: from the cradle of evolution in Africa to Eurasia, Australia, and the Americas. *J. Virol.* 85 2964–2974. 10.1128/JVI.01963-10 21159871 PMC3067944

[B88] Montgomery S. P. Brown J. A. Kuehnert M. Smith T. L. Crall N. Lanciotti R. S. (2006). Transfusion-associated transmission of West Nile virus, United States 2003 through 2005. *Transfusion* 46 2038–2046. 17176314 10.1111/j.1537-2995.2006.01030.x

[B89] Morelli M. C. Sambri V. Grazi G. L. Gaibani P. Pierro A. Cescon M. (2010). Absence of neuroinvasive disease in a liver transplant recipient who acquired West Nile virus (WNV) infection from the organ donor and who received WNV antibodies prophylactically. *Clin. Infect. Dis.* 51 e34–e37. 10.1086/655146 20597692

[B90] Mulatti P. Bonfanti L. Capelli G. Capello K. Lorenzetto M. Terregino C. (2013). West Nile virus in north-eastern Italy, 2011: entomological and equine IgM-based surveillance to detect active virus circulation. *Zoon. Public Health* 60 375–382. 10.1111/zph.12013 22971022

[B91] Murray K. Walker C. Herrington E. Lewis J. A. McCormick J. Beasley D. W. (2010). Persistent infection with West Nile virus years after initial infection. *J. Infect. Dis.* 201 2–4. 10.1086/648731 19961306 PMC2791189

[B92] Murray K. O. Garcia M. N. Yan C. Gorchakov R. (2013). Persistence of detectable immunoglobulin M antibodies up to 8 years after infection with West Nile virus. *Am. J. Trop. Med. Hyg.* 89 996–1000. 10.4269/ajtmh.13-0232 24062481 PMC3820351

[B93] Myhrvold C. Freije C. A. Gootenberg J. S. Abudayyeh O. O. Metsky H. C. Durbin A. F. (2018). Field-deployable viral diagnostics using CRISPR-Cas13. *Science* 360 444–448. 10.1126/science.aas8836 29700266 PMC6197056

[B94] Napoli C. Bella A. Declich S. Grazzini G. Lombardini L. Nanni Costa A. (2013). Integrated human surveillance systems of West Nile virus infections in Italy: the 2012 experience. *Int. J. Environ. Res. Public Health* 10 7180–7192. 10.3390/ijerph10127180 24351740 PMC3881160

[B95] Nash D. Mostashari F. Fine A. Miller J. O’Leary D. Murray K. (2001). The outbreak of West Nile virus infection in the New York City area in 1999. *N. Engl. J. Med.* 344 1807–1814. 11407341 10.1056/NEJM200106143442401

[B96] Nelson S. Jost C. A. Xu Q. Ess J. Martin J. E. Oliphant T. (2008). Maturation of West Nile virus modulates sensitivity to antibody-mediated neutralization. *PLoS Pathog.* 4:e1000060. 10.1371/journal.ppat.1000060 18464894 PMC2330159

[B97] Orshan L. Bin H. Schnur H. Kaufman A. Valinsky A. Shulman L. (2008). Mosquito vectors of West Nile Fever in Israel. *J. Med. Entomol.* 45 939–947. 18826039 10.1603/0022-2585(2008)45[939:mvownf]2.0.co;2

[B98] Pai A. Kleinman S. Malhotra K. Lee-Haynes L. Pietrelli L. Saldanha J. (2008). Performance characteristics of the Food and Drug Administration-licensed Roche Cobas TaqScreen West Nile virus assay. *Transfusion* 48 2184–2189. 10.1111/j.1537-2995.2008.01861.x 18694466

[B99] Papa A. (2012). West Nile virus infections in Greece: an update. *Expert. Rev. Anti Infect. Ther.* 10 743–750. 10.1586/eri.12.59 22943398

[B100] Papa A. Anastasiadou A. Delianidou M. (2015). West Nile virus IgM and IgG antibodies three years post- infection. *Hippokratia* 19 34–36. 26435644 PMC4574583

[B101] Papa A. Danis K. Athanasiadou A. Delianidou M. Panagiotopoulos T. (2011). Persistence of West Nile virus immunoglobulin M antibodies, Greece. *J. Med. Virol.* 83 1857–1860. 10.1002/jmv.22190 21837805

[B102] Papa A. Danis K. Baka A. Bakas A. Dougas G. Lytras T. (2010). Ongoing outbreak of West Nile virus infections in humans in Greece, July-August 2010. *Euro. Surveill.* 15:19644. 20807489 10.2807/ese.15.34.19644-en

[B103] Paternoster G. Babo Martins S. Mattivi A. Cagarelli R. Angelini P. Bellini R. (2017a). Economics of One Health: costs and benefits of integrated West Nile virus surveillance in Emilia-Romagna. *PLoS One* 12:0188156. 10.1371/journal.pone.0188156 29176851 PMC5703535

[B104] Paternoster G. Tomassone L. Tamba M. Chiari M. Lavazza A. Piazzi M. (2017b). The Degree of One Health Implementation in the West Nile Virus Integrated Surveillance in Northern Italy, 2016. *Front. Public Health* 5:236. 10.3389/fpubh.2017.00236 28929098 PMC5591825

[B105] Patsoula E. Vakali A. Balatsos G. Pervanidou D. Beleri S. Tegos N. (2016). West Nile Virus Circulation in Mosquitoes in Greece (2010-2013). *Biomed.Res.Int* 2016:2450682. 10.1155/2016/2450682 27294111 PMC4880692

[B106] Paz S. Semenza J. C. (2013). Environmental drivers of West Nile fever epidemiology in Europe and Western Asia–a review. *Int. J. Environ. Res. Public Health* 10 3543–3562. 10.3390/ijerph10083543 23939389 PMC3774453

[B107] Pealer L. N. Marfin A. A. Petersen L. R. Lanciotti R. S. Page P. L. Stramer S. L. (2003). Transmission of West Nile virus through blood transfusion in the United States in 2002. *N. Engl. J. Med.* 349 1236–1245. 14500806 10.1056/NEJMoa030969

[B108] Pervanidou D. Detsis M. Danis K. Mellou K. Papanikolaou E. Terzaki I. (2014). West Nile virus outbreak in humans, Greece, 2012: third consecutive year of local transmission. *Euro. Surveill.* 19:20758 24721540 10.2807/1560-7917.es2014.19.13.20758

[B109] Petersen L. R. Brault A. C. Nasci R. S. (2013). West Nile virus: review of the literature. *JAMA* 310 308–315. 10.1001/jama.2013.8042 23860989 PMC4563989

[B110] Pezzotti P. Piovesan C. Barzon L. Cusinato R. Cattai M. Pacenti M. (2011). Prevalence of IgM and IgG antibodies to West Nile virus among blood donors in an affected area of north-eastern Italy, summer 2009. *Euro. Surveill.* 16:19814 21435323 10.2807/ese.16.10.19814-en

[B111] Pierro A. Gaibani P. Manisera C. Dirani G. Rossini G. Cavrini F. (2011). Seroprevalence of West Nile virus-specific antibodies in a cohort of blood donors in northeastern Italy. *Vector Borne Zoon. Dis.* 11 1605–1607. 10.1089/vbz.2011.0616 21867418

[B112] Pierro A. Gaibani P. Spadafora C. Ruggeri D. Randi V. Parenti S. (2013). Detection of specific antibodies against West Nile and Usutu viruses in healthy blood donors in northern Italy, 2010-2011. *Clin. Microbiol. Infect.* 19 E451–E453. 10.1111/1469-0691.12241 23663225

[B113] Pisani G. Cristiano K. Pupella S. Liumbruno G. M. (2016). West Nile Virus in Europe and Safety of Blood Transfusion. *Transfus Med. Hemother.* 43 158–167. 10.1159/000446219 27403087 PMC4924479

[B114] Poh K. C. Martin E. Walker E. D. Kitron U. Ruiz M. O. Goldberg T. L. (2018). Co-circulation of Flanders Virus and West Nile Virus in Culex Mosquitoes (Diptera: Culicidae) from Chicago, Illinois. *J. Med. Entomol.* 55 1062–1066. 29659921 10.1093/jme/tjy051 PMC6025230

[B115] Prince H. E. Tobler L. H. Lape-Nixon M. Foster G. A. Stramer S. L. Busch M. P. (2005). Development and persistence of West Nile virus-specific immunoglobulin M (IgM), IgA, and IgG in viremic blood donors. *J. Clin. Microbiol.* 43 4316–4320. 16145071 10.1128/JCM.43.9.4316-4320.2005 PMC1234148

[B116] Rabel P. O. Planitzer C. B. Farcet M. R. Orlinger K. K. Ilk R. Barrett P. N. (2011). Increasing West Nile virus antibody titres in central European plasma donors from 2006 to 2010. *Euro. Surveill.* 16. 21435324 10.2807/ese.16.10.19812-en

[B117] Ramamurthy M. Sankar S. Kannangai R. Nandagopal B. Sridharan G. (2017). Application of viromics: a new approach to the understanding of viral infections in humans. *Virusdisease* 28 349–359. 10.1007/s13337-017-0415-3 29291225 PMC5747850

[B118] Rappole J. H. Hubalek Z. (2003). Migratory birds and West Nile virus. *J. Appl. Microbiol.* 94 (Suppl.), 47S–58S. 12675936 10.1046/j.1365-2672.94.s1.6.x

[B119] Reisen W. K. Takahashi R. M. Carroll B. D. Quiring R. (2008). Delinquent mortgages, neglected swimming pools, and West Nile virus, California. *Emerg. Infect. Dis.* 14 1747–1749. 10.3201/eid1411.080719 18976560 PMC2630753

[B120] Rios M. Daniel S. Chancey C. Hewlett I. K. Stramer S. L. (2007). West Nile virus adheres to human red blood cells in whole blood. *Clin. Infect. Dis.* 45 181–186. 17578776 10.1086/518850

[B121] Rizzo C. Napoli C. Venturi G. Pupella S. Lombardini L. Calistri P. (2016). West Nile virus transmission: results from the integrated surveillance system in Italy, 2008 to 2015. *Euro. Surveill.* 21:30340. 27684046 10.2807/1560-7917.ES.2016.21.37.30340 PMC5032855

[B122] Roehrig J. T. (2013). West nile virus in the United States - a historical perspective. *Viruses* 5 3088–3108. 10.3390/v5123088 24335779 PMC3967162

[B123] Rosa R. Marini G. Bolzoni L. Neteler M. Metz M. Delucchi L. (2014). Early warning of West Nile virus mosquito vector: climate and land use models successfully explain phenology and abundance of Culex pipiens mosquitoes in north-western Italy. *Parasit Vectors* 7:269. 10.1186/1756-3305-7-269 24924622 PMC4061321

[B124] Ruiz M. O. Walker E. D. Foster E. S. Haramis L. D. Kitron U. D. (2007). Association of West Nile virus illness and urban landscapes in Chicago and Detroit. *Int. J. Health Geogr.* 6:10. 17352825 10.1186/1476-072X-6-10 PMC1828048

[B125] Salama M. Amitai Z. Lustig Y. Mor Z. Weiberger M. Chowers M. (2018). Outbreak of West Nile Virus disease in Israel (2015): a retrospective analysis of notified cases. *Travel Med.Infect.Dis.* 10.1016/j.tmaid.2018.07.008 [Epub ahead of print]. 30016649

[B126] Sanchez-Gomez A. Amela C. Fernandez-Carrion E. Martinez-Aviles M. Sanchez-Vizcaino J. M. Sierra-Moros M. J. (2017). Risk mapping of West Nile virus circulation in Spain, 2015. *Acta Trop.* 169 163–169. 10.1016/j.actatropica.2017.02.022 28212847

[B127] Seino K. K. Long M. T. Gibbs E. P. Bowen R. A. Beachboard S. E. Humphrey P. P. (2007). Comparative efficacies of three commercially available vaccines against West Nile Virus (WNV) in a short-duration challenge trial involving an equine WNV encephalitis model. *Clin. Vaccine Immunol.* 14 1465–1471. 17687109 10.1128/CVI.00249-07 PMC2168174

[B128] Simeone R. M. Shapiro-Mendoza C. K. Meaney-Delman D. Petersen E. E. Galang R. R. Oduyebo T. (2016). Possible Zika Virus Infection Among Pregnant Women - United States and Territories, May 2016. *MMWR Morb. Mortal. Wkly. Rep.* 65 514–519. 10.15585/mmwr.mm6520e1 27248295

[B129] Song B. H. Yun S. I. Woolley M. Lee Y. M. (2017). Zika virus: history, epidemiology, transmission, and clinical presentation. *J. Neuroimmunol.* 308 50–64. 10.1016/j.jneuroim.2017.03.001 28285789

[B130] Steiner I. Kennedy P. G. (2013). West Nile virus introduction into the New World. *Neurology* 81 1441–1442. 24127189 10.1212/WNL.0b013e3182a840ad

[B131] Steinhagen K. Probst C. Radzimski C. Schmidt-Chanasit J. Emmerich P. van Esbroeck M. (2016). Serodiagnosis of Zika virus (ZIKV) infections by a novel NS1-based ELISA devoid of cross-reactivity with dengue virus antibodies: a multicohort study of assay performance, 2015 to 2016. *Euro. Surveill.* 21:30426. 28006649 10.2807/1560-7917.ES.2016.21.50.30426 PMC5291135

[B132] Stiasny K. Aberle S. W. Heinz F. X. (2013). Retrospective identification of human cases of West Nile virus infection in Austria (2009 to 2010) by serological differentiation from Usutu and other flavivirus infections. *Euro. Surveill.* 18:20614. 24176619 10.2807/1560-7917.es2013.18.43.20614

[B133] Tesh R. B. Parsons R. Siirin M. Randle Y. Sargent C. Guzman H. (2004). Year-round West Nile virus activity, Gulf Coast region, Texas and Louisiana. *Emerg. Infect. Dis.* 10 1649–1652. 15498169 10.3201/eid1009.040203 PMC3320313

[B134] Turell M. J. Dohm D. J. Sardelis M. R. Oguinn M. L. Andreadis T. G. Blow J. A. (2005). An update on the potential of north American mosquitoes (Diptera: culicidae) to transmit West Nile Virus. *J. Med. Entomol.* 42 57–62. 15691009 10.1093/jmedent/42.1.57

[B135] Valiakos G. Papaspyropoulos K. Giannakopoulos A. Birtsas P. Tsiodras S. Hutchings M. R. (2014). Use of wild bird surveillance, human case data and GIS spatial analysis for predicting spatial distributions of West Nile virus in Greece. *PLoS One* 9:e96935. 10.1371/journal.pone.0096935 24806216 PMC4013071

[B136] Vanlandingham D. L. McGee C. E. Klinger K. A. Vessey N. Fredregillo C. Higgs S. (2007). Relative susceptibilties of South Texas mosquitoes to infection with West Nile virus. *Am. J. Trop. Med. Hyg.* 77 925–928. 17984355

[B137] Vrioni G. Mavrouli M. Kapsimali V. Stavropoulou A. Detsis M. Danis K. (2014). Laboratory and clinical characteristics of human West Nile virus infections during 2011 outbreak in southern Greece. *Vector Borne Zoon. Dis.* 14 52–58. 10.1089/vbz.2013.1369 24359426

[B138] Weinberger M. Pitlik S. D. Gandacu D. Lang R. Nassar F. Ben David D. (2001). West Nile fever outbreak, Israel, 2000: epidemiologic aspects. *Emerg. Infect. Dis.* 7 686–691. 11585533 10.3201/eid0704.010416 PMC2631774

[B139] White D. J. Kramer L. D. Backenson P. B. Lukacik G. Johnson G. Oliver J. A. (2001). Mosquito surveillance and polymerase chain reaction detection of West Nile virus, New York State. *Emerg. Infect. Dis.* 7 643–649. 11585526 10.3201/eid0704.010407 PMC2631741

[B140] Williams S. H. Cordey S. Bhuva N. Laubscher F. Hartley M. A. Boillat-Blanco N. (2018). Investigation of the Plasma Virome from Cases of Unexplained Febrile Illness in Tanzania from 2013 to 2014: a Comparative Analysis between Unbiased and VirCapSeq-VERT High-Throughput Sequencing Approaches. *mSphere* 3: e00311-18. 10.1128/mSphere.00311-18 PMC6106054 30135221

[B141] Wilson M. R. Zimmermann L. L. Crawford E. D. Sample H. A. Soni P. R. Baker A. N. (2017). Acute west nile virus meningoencephalitis diagnosed via metagenomic deep sequencing of cerebrospinal fluid in a renal transplant patient. *Am. J. Transplant.* 17 803–808. 10.1111/ajt.14058 27647685 PMC5347949

[B142] Zana B. Kemenesi G. Herczeg R. Dallos B. Oldal M. Marton S. (2016). Genomic characterization of West Nile virus strains derived from mosquito samples obtained during 2013 Serbian outbreak. *J. Vector Borne Dis.* 53 379–383. 28035117

[B143] Zhang W. Wu J. Li Y. Li F. Njoo H. (2009). Rapid and accurate in vitro assays for detection of West Nile virus in blood and tissues. *Transfus Med. Rev.* 23 146–154. 10.1016/j.tmrv.2008.12.008 19304115

[B144] Zheng H. Drebot M. A. Coulthart M. B. (2014). West Nile virus in Canada: ever-changing, but here to stay. *Can. Commun. Dis. Rep.* 40 173–177. 29769840 10.14745/ccdr.v40i10a01 PMC5864456

[B145] Ziermann R. Sanchez-Guerrero S. A. (2008). PROCLEIX West Nile virus assay based on transcription-mediated amplification. *Expert Rev. Mol. Diagn.* 8 239–245. 10.1586/14737159.8.3.239 18598103

